# Morphometric Analysis of the Midline Mandibular Lingual Canal and Mandibular Lingual Foramina: A Cone Beam Computed Tomography (CBCT) Evaluation

**DOI:** 10.3390/ijerph192416910

**Published:** 2022-12-16

**Authors:** Ahmed Yaseen Alqutaibi, Muath Saad Alassaf, Shadia A. Elsayed, Abdulmajeed Saud Alharbi, Abdulsamad Talaat Habeeb, Marwan Ahmad Alqurashi, Khalid Ahmed Albulushi, Mohamed Omar Elboraey, Kamal Alsultan, Ihab Ismail Mahmoud

**Affiliations:** 1 Prosthodontics, and Dental Implant Department, College of Dentistry, Taibah University, Al Madinah 41311, Saudi Arabia; 2 Prosthodontics Department, College of Dentistry, Ibb University, Ibb 70270, Yemen; 3 Dental College and Hospital, Taibah University, Al Madinah 41311, Saudi Arabia; 4 Oral and Maxillofacial Surgery, College of Dentistry, Taibah University, Al Madinah 41311, Saudi Arabia; 5 Oral and Maxillofacial Surgery, Faculty of Dental Medicine for Girls, AL-Azhar University, Cairo 11884, Egypt; 6 Oral Medicine, Periodontology, Oral Diagnosis and Radiology Department, Faculty of Dentistry, Tanta University, Tanta 31527, Egypt; 7 Periodontology Department, College of Dentistry, Taibah University, Al Madinah 41311, Saudi Arabia; 8 Diagnostic Radiology Technology, College of Applied Medical Sciences, Taibah University, Al Madinah 41311, Saudi Arabia; 9 Removable Prosthodontics, Faculty of Dental Medicine, Al-Azhar University, Cairo 11884, Egypt

**Keywords:** CBCT, prevalence, lingual foramen, mandibular lingual canal, mandible

## Abstract

Background: This study aimed to evaluate the midline mandibular lingual canals and foramina and their anatomic variations using CBCT scans. Methods: This study used retrospective analysis. A total of 320 CBCT scans were used to evaluate the study parameters, which comprised the presence or absence of the mandibular lingual foramen (MLF)/mandibular lingual canal (MLC) and its category, the distance between the buccal cortex and the start of the MLC, the distance between the inferior border of the mandible and the superior border of the foramen at its lingual and buccal terminals. The length and diameter of each canal at its lingual and buccal terminals. Results: MLC was found in all included CBCT scans. Out of 320 included CBCT scans, a single canal was represented by 30.9%, double canals (Supra with Infra -spinosum) configuration appeared in 54.7%, and triple canals (Supra-Inter-Infra) represented 14.7%. The supraspinosum canals averaged 5.81 ± 2.08 mm in length and 0.87 ± 0.30 mm in diameter at the lingual terminal. In terms of the number of canals, there was a significant difference between men and women (*p* ≤ 0.001), with 60% of the men in the sample having double canals and 43.1% of the women having single canals. Moreover, the male gender had a higher prevalence of triple canals (21.3% vs. 8.1%) than females. Males and females were distributed equally among the supraspinosum canals, with no statistically significant difference (*p* ≤ 0.7). A considerable increase in the finding of interspinosum and infraspinosum canals was seen in the male sample (*p* ≤ 0.001). Conclusions: midline mandibular canals were found in all investigated CBCTs of the sample of both sexes; however, the anatomy and location of the MLF and canals varied significantly among the Saudi population.

## 1. Introduction

The anatomy of the midline lingual canal (MLC) of the mandible and its lingual foramen (MLF) is of great importance for planning for midline dental implant placement, donor site for block bone graft, and other surgeries involving the anterior mandible [1,2,3,4]. The midline lingual mandibular foramen and its bony canal are located at the internal surface of the anterior region of the mandible; according to cadaver studies, branches from the sublingual and submental arteries go through these anatomic structures [5,6,7]. The arteries anastomose beneath the mucous membrane of the anterior floor of the mouth and penetrate the lingual cortical of the mandible through the MLF, thus anastomosing with the central alveolar vessels [7].

Although the mandibular midline area is believed to be safe for any surgical intervention, violation of this canal may lead to severe life-threatening complications such as severe bleeding and airway obstruction if the content of the MLC is injured. Among the reported complications are severe bleeding and airway obstruction during midline implant placement [8].

Several studies examined the anatomic variations of the MLC in different populations; in contrast to several reports [9,10,11,12] that showed the absence of this anatomical structure in some of the examined populations, other studies [13,14,15] revealed that the prevalence of MLC and its foramen is 100%. A recent systematic review and meta-analysis of 8255 CBCT scans from 18 different countries reported that the overall prevalence of the MLC was 90.11%, with great variations between different nationalities [16]. So, the prevalence differs from country to country, emphasizing the importance of conducting such a study in Saudi Arabia to fill this population knowledge gap. It is worth noting that research into this anatomic structure is critical in oral and maxillofacial surgery, including the surgical phase of the dental implant.

Possible variations in location, number, and length of MLC make detection difficult with conventional radiographic evaluation; therefore, there is a need for three-dimensional visualization of the mandible using imaging technology that provides a detailed image of the bone architecture with great contrast as CBCT [17,18].

This study aims to evaluate the midline mandibular lingual canals and foramina and their anatomic variations using CBCT scans of the Saudi population sample.

## 2. Materials and Methods

### 2.1. Subjects

This retrospective study screened scans from patients who had visited Taibah University Dental Hospital (TUDH) and had undergone CBCT imaging between 2018 and 2022. CBCT scans of Saudi adult patients between 16 and 72 years were included. Patients with a history of trauma, pathology, surgical intervention congenital syndrome, fracture, or any other foreign body (which produced artifacts in the image) in the area between the two mental foramina were excluded from the study. Moreover, scans not containing an area of interest (i.e., midline of the mandible), blurred scans, such as those containing an ill-defined and unclear definition of bony borders, were excluded from the study. Of 1080 screened scans, 534 were excluded according to the abovementioned criteria. Of the remaining 546 (310 male, 236 female) scans that fulfilled the eligibility criteria, 320 of the CBCT scans were chosen by using a spreadsheet (Excel; Microsoft Corp., Redmond, WA, USA) to generate random numbers ensuring male to female ratio of 1:1. The sample size was calculated using parameters that closely matched the Saudi population: 80% power, a 95% confidence interval, and a 0.05 margin of error. Anonymous code was given to each subject, all the data was saved in an excel sheet with a security password, and the computer was secured with a password. The Research Ethical Committee of the College of Dentistry, Taibah University, Madinah, Saudi Arabia, approved this study (approval # 04042022).

### 2.2. Data Collection, Image Reconstruction, and Assessments

The CBCT machine (KaVo 3D eXam; KaVo) in the TUDH obtained all the CBCT scans. This retrospective study used CBCT scans already taken for cases requiring additional three-dimensional imaging evaluation for diagnosis and treatment purposes. All scans were made at 120 kVp and 5 mA using a field of view of 16 × 13 cm, 26.9 s of acquisition time, and a voxel size of 0.25 mm. Every CBCT scan performed in the TUDH adhered to a standardized scanning protocol. Patients have been positioned with the machine head-chin positioner. Radiographers instructed all patients to stay motionless during the scan.

**Figure 1:**
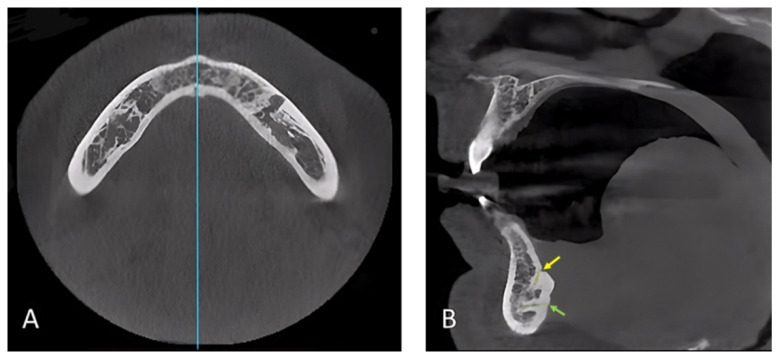
CBCT scan orientation. (**A**) Axial cut at the level of the genial tubercle to orient the midline. (**B**) Sagittal cut at the midline showing the mandibular lingual canals. The yellow arrow points to the supra-spinosum foramen, which is the opening of its corresponding canal (i.e., yellow line). The green arrow points to the infra-spinosum foramen, which is the opening of its corresponding canal (i.e., green line).

**Figure 2:**
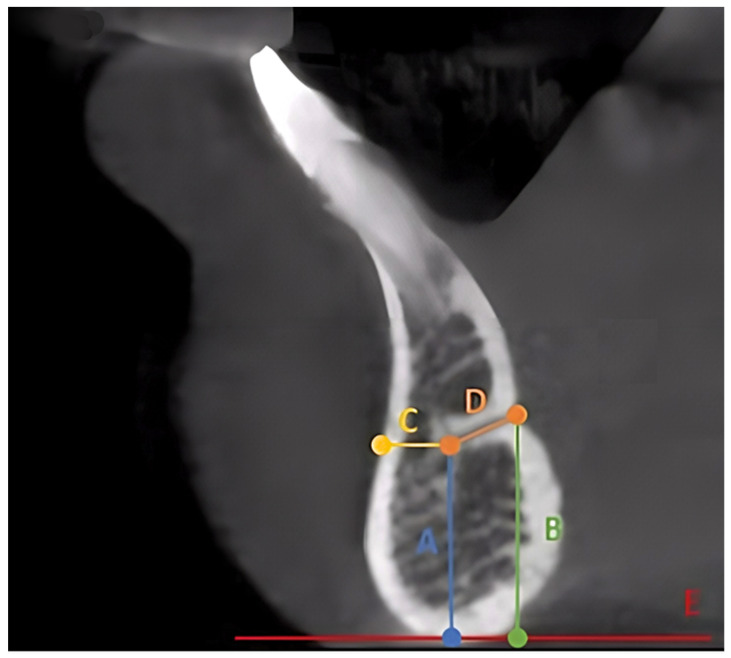
CBCT sagittal cut photograph showing study variable measurements: (A) Inferior border to the buccal terminal; (B) inferior border to the lingual terminal; (C) buccal cortex to the buccal terminal; (D) canal length; (E) lower border reference line.

All scans were analyzed using RadiAnt DICOM Viewer (Version 2022.1.1) on a computer screen. Two mandibular cuts were used to collect the data (Figure 1), an axial cut at the level of the genial spines to orient the midline and a sagittal cut at the level oriented by the axial cut. Then three slices of 250 microns thickness were used to assess the following study variables (Figure 2): Presence or absence of the MLF/MLC and its category; the distance from the inferior border of the mandible to the superior border of the foramen at its lingual and buccal terminals; the inferior border is used to measure the distance because it is more consistent and has fewer changes compared to the crestal bone; the distance from the buccal cortex to the beginning of the MLC; the length of the canal or canals; the diameter at the lingual and buccal terminals for each canal. All are measured in millimeters. The canal direction is determined by drawing a line connecting the buccal and lingual terminals and then comparing this line to the line at the inferior border; when these lines are parallel, the canal is considered straight. If diverging, the canal is directed upward, or if converging, the canal is directed downward.

Two investigators with more than ten years of expertise in CBCT analysis and interpretation shared software instructions and repeated suitable manipulations to ensure effective data-collecting calibration and standardize the reading and interpretation. Prior to the actual CBCT scanevaluation, each examiner evaluated ten CBCT scans; these scans were selected from surplus data and were not included in these study results. Without access to the first data, the same examiners took the second action to identify errors. In a disagreement, the mean of the two values was considered. Intra-examiner and inter-examiner testing were done.

The MLF has no standard classification approach; however, in this study, the MLF/MLC will be described as supraspinosum and infraspinosum foramen according to its relation to the genial tubercle (S-MLF and I-MLF). Their canals are named supraspinosum and infraspinosum MLC (S-MLC and I-MLC).

### 2.3. Statistical Analysis

The Statistical Package for Social Science 23 (SPSS, version 23, Inc., Chicago, IL, USA) was used for data analysis. Descriptive analysis was used to summarize the sample characteristics. The continuous variables were presented as mean with standard deviations (M ± SD) if data were normally distributed (Kolmogorov–Smirnov *p* > 0.05); if not normally distributed, the median with the interquartile range (IQR) was used. Qualitative variables were analyzed as frequency and percentages. Pearson correlation was utilized to assess interrater reliability. Appropriate parametric tests such as Student or paired *t*-test were used for normally distributed data, and non-parametric tests such as the U-test were used if data were not normally distributed. The chi-square test was used to compare between groups for qualitative variables. The significance level was set at 5%.

## 3. Results

This study comprised 320 CBCT scans from 160 men (50%) and 160 women (50%). The patients ranged in age from 16 to 72 years old, with a mean age of 41 ± 14.2. The age distribution was comparable across the male and female genders (*p* > 0.05).

MLC was identified in all cases. Concerning the number of lingual foramina, out of 320 included CBCT scans, a single canal was represented by 30.9%, double canals (Supra with Infra -spinosum) configuration appeared in 54.7%, and triple canals (Supra–Inter-Infra) represented 14.7%.

**Figure 3:**
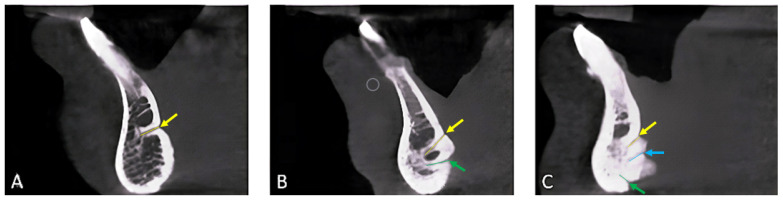
Sagittal view at the mandibular lingual canal/s of CBCT scan showing three different variations, (**A**) single, (**B**) double, (**C**) triple canals. The yellow arrow points to the supra-spinosum foramen, which is the opening of its corresponding canal (i.e., yellow line). The blue arrow points to the inter-spinosum foramen, which is the opening of its corresponding canal (i.e., blue line). The green arrow points to the infra-spinosum foramen, which is the opening of its corresponding canal (i.e., green line).

In relation to position, most of the lingual foramina (305 [95.3%]) were positioned superior to the genial tubercle (i.e., supraspinosum MLC), with the majority (97.7%) directed upward. There were 47 interspinosum canals, which were not found alone and were always associated with supra or infraspinosum canals, most of which were directionally straight (48.9%). There were 237 infraspinosum canals, most oriented downward (71.7%) (Table 1). The three variations (i.e., single, double, and triple canals) that appeared in the investigated sample are shown in Figure 3.

A descriptive analysis of the studied cases according to the position, length, and diameter of MLCs is shown in Table 2. The supraspinosum canals had the greatest lengths and the broadest diameters at the lingual terminals (5.81± 2.08 and 0.87± 0.30, respectively).

In terms of the number of canals, there was a significant difference between males and females (*p* ≤ 0.001), with 60% of the male in the sample having double canals and 43.1% of the females having single canals. Additionally, triple canal frequency was higher in males than in females (21.3% vs. 8.1%). Males and females were distributed equally among the supraspinosum canals, with no statistically significant difference (*p* = 0.7); a significant prevalence of interspinosum and infraspinosum canals was seen in the male sample (*p* ≤ 0.001) (Table 3).

Comparison between males and females according to study variables measurements are presented in Table 4. The male subjects showed significantly greater distances from the inferior border to the buccal terminal and lingual terminals of the MLC. Similarly, the length of supra-spinosum MLC was significantly longer in males than in females, and the male subjects showed statistically significant wider canal diameters from both ends (*p* < 0.001) (Table 4).

**Figure 4:**
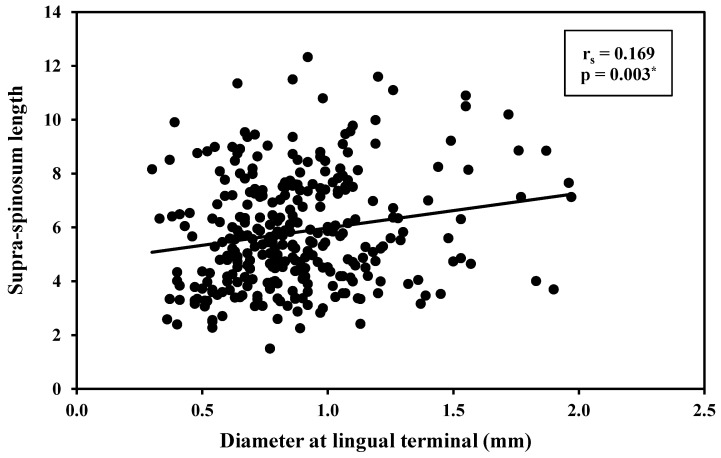
Correlation between supra-spinosum canal length and its diameter at the lingual terminal (mm). r_s_: Spearman coefficient; *: statistically significant at *p* ≤ 0.05.

The association of canal length (mm) versus its diameter at the lingual terminal found that as the canal increased in length, it had wider foramen (Table 5); however, only the supraspinosum canals had a statistically significant correlation between canal length and diameter (r = 0.169, *p* = 0.003) (Figure 4).

The Pearson correlation was utilized to assess interrater reliability by comparing the measurements of both raters. The findings demonstrated a reliability of 86.4%, indicating a high level of agreement in both raters’ readings.

## 4. Discussion

Individuals differ significantly regarding the MLF/MLC position, size, frequency, and other characteristics. The anatomy of these structures was evaluated using various techniques, including cadaver studies, CBCT, and ultrasound [7,15,19].

Previously, oral surgeons relied on lateral cephalometric and panoramic radiographs to examine the lingual cortex of the mandible; however, these modalities often fail to indicate this anatomical structure’s visibility and features because of superimposition and geometric distortion [20,21]. This study used CBCT to collect data about the mandibular lingual canal in a cohort of Saudi residents. CBCT is more accurate and can effectively present the mandibular lingual foramen and associated bony canal changes. The image quality of CBCT systems and their reduced dosage and cost compared to traditional computed tomography have made the three-dimensional examination of craniofacial features more accessible in dental practice [11]. The visualization of the MLF/MLC depends on technological parameters such as image resolution and reconstruction time of the 3D scan. In our study, all CBCT scans were made at the same specific machine settings (e.g., scan mode, kV, voxel size rotation degree, the field of view, and mA); as the change in any of these factors may influence the visualization of the anatomical structures [22].

From a practical standpoint, dentists should be familiar with terminology related to MLF. In the published literature, this anatomical structure is also known as midline lingual foramen, medial lingual canal, lingual vascular canal, superior or inferior genial spinal foramina (according to its relation to the genial spines); sometimes it is described as foramen inter-spinosum (when it is at the level of the genial spines), mental spinal foramina, mandibular accessory foramina, lingual foramina, or lingual accessory mental foramen [19,23,24,25].

The primary study finding indicated that at least one MLF/MLC was present in all samples, which was in accordance with other studies [13,14,15]. The results of a recent systematic review conducted by Barbosa et al. [16] found that these canals were more prevalent in females than males, which contradicts our findings, which showed an insignificant difference between males and females regarding the presence of these anatomical structures, and this may be due to the diversity of the populations studied, as they included studies from Asia, Europe, North America, and South America.

In the present study, the supraspinosum canals were the most commonly present, and interspinosum canals were always in combination with supraspinosum and infraspinosum canals; however, the interspinosum canals were never present alone.

Although other studies have found up to four MLFs [5,26,27], the most common presentation of the mandibular lingual canals in this study was a double canal (i.e., supra with infraspinosum) followed by a single canal, and the presence of three canals was scarce. This finding is consistent with other studies that have found the most common number to be two, and single MLF are frequently found above the genial spines [7,28,29]. Moreover, our finding revealed the SMLF diameter at its lingual end was 0.87 ± 0.30, with men tending to have wider canals than women, which goes in accordance with the study conducted by Bulut and Köse [2].

The important clinical issue connected to the number of lingual foramina and their diameter is the probability of severe hemorrhagic episodes that might occur during or following surgical intervention in the anterior mandibular [30]. The sublingual artery is an important branch of the lingual artery and nourishes this anatomical region through intraosseous channels; a higher number of lingual foramina indicates increased vascularization. Furthermore, the wider the MLC, the more calibrated the vessel that flows inside [30].

Bone graft collection procedures in the anterior region of the mandible are commonly performed in clinical practice, so adequate buccal bone cortical limits should be rigorously planned in surgical procedures involving bone graft insertion. Our finding revealed the distances from the SMLF to the buccal bone cortex of 5.84 ± 1.7mm, and these results were comparable to other studies [15,18].

Sheiki et al. and Liang reported average S-MLC lengths of 7.83 ± 2.25 mm and 6.8 ± 2.3 mm, respectively. These values are slightly longer than the current study results since the supraspinosum canals had an average length of 5.81 ± 2.08 mm. This may be due to the diverse populations studied [5,15].

Most of the patients in the current study (98.4%) were dentate, which coincides with other studies [31]. More research should be conducted in our region to distinguish between edentulous and dentate positions and the anatomical variances of the mandibular lingual canals.

The current study results are beneficial to all implantologists in the region of Saudi Arabia because of the high frequency of mandibular canals there, and they should warn them of the potential complications such as bleeding and hematoma formation that can occur during implant insertion close to the mandibular midline region [23].

All surgeons and implantologists should not minimize the risk of life-threatening situations resulting from surgical procedures, including dental implant placement in an apparently safe region of the mandible, especially when they know that MLF/MLC frequency is high and complications may occur [8,25,32]. Therefore, it is essential to stress the following advice. First, before performing surgery on the anterior mandible, precise preoperative planning using CBCT is necessary to prevent unusual complications that accurately consider the degree of bony atrophy and mandibular inclination. Second, to prevent accidental lacerations, adequate operator surgical training in lingual mucoperiosteal flap preparation maneuvers, especially in elderly patients is required.

There are several limitations to this study that should be mentioned. Despite the fact that this study was conducted in a single center (Taibah University Dental Hospital, Medina, Saudi Arabia), the sample is likely to represent the Saudi population. Notably, Medina city is resided and commuted to by many residents from various regions of Saudi Arabia. However, future research should consider conducting this study in a multicenter setting. It is worth noting that the results of this study cannot be applied to the whole world’s population because of racial and ethnic variances; in addition, the scope of this research was limited to the bone canals on the lingual side of the mandibular midline. Future studies must investigate the frequency and anatomical differences of the midline or lateral lingual foramina and canals across the many ethnic groups that comprise the global people, considering the effect of different parameters (e.g., age and BMI) on the characteristics of these anatomical structures.

## 5. Conclusions

Mandibular canals were found in all investigated CBCTs of the sample of both sexes; however, the anatomy and location of the MLF and canals varied significantly among the Saudi population. The oral surgeon should know this anatomic feature and its possible implications. The anatomy of the mandibular lingual canal and its foramina is crucial for planning dental implants and other surgical procedures involving the anterior mandible to avoid severe life-threatening complications.

## Figures and Tables

**Table 1:** Distribution of the studied cases according to supra, inter, and infra-spinosum (*n* = 320).

	No.	%
Supra-spinosum only	83	25.9
Infra-spinosum only	15	4.7
Supra with Inter-spinosum	0	0.0
Supra with Infra -spinosum	175	54.7
Supra-Inter-Infra together	47	14.7

**Table 2:** Descriptive analysis of the studied cases according to position, length, and diameter.

Variable	Category	Min.–Max.	Mean ± SD	Median (IQR)
Inferior border to buccal terminal (mm)	supra-spinosum	5.30–17.75	11.03 ± 2.40	11.18 (9.3–12.67)
	inter-spinosum	3.68–13.85	8.26 ± 2.16	8.17 (6.60–9.91)
	infra-spinosum	0.53–12.55	6.44 ± 2.13	6.42 (4.99–7.73)
Inferior border to lingual terminal (mm)	supra-spinosum	6.86–20.05	14.95 ± 2.19	15.15 (13.6–16.4)
	inter-spinosum	2.97–13.85	8.70 ± 2.42	8.49 (7.77–10.26)
	infra-spinosum	0.58–14.15	5.25 ± 2.71	4.78 (3.36–6.73)
Buccal cortex to buccal terminal (mm)	supra-spinosum	1.66–10.50	5.84 ± 1.69	5.76 (4.53–6.96)
	inter-spinosum	2.75–9.58	5.96 ± 1.70	5.84 (4.61–7.12)
	infra-spinosum	1.54–13.0	5.81 ± 1.76	5.65 (4.44–6.86)
Length (mm)	supra-spinosum	1.50–12.33	5.81 ± 2.08	5.54 (4.15–7.36)
	inter-spinosum	2.41–9.39	5.52 ± 1.75	4.98 (4.12–7.0)
	infra-spinosum	1.35–9.53	5.16 ± 1.86	4.91 (3.65–6.61)
Diameter at buccal terminal (mm)	supra-spinosum	0.22–1.76	0.60 ± 0.20	0.59 (0.48–0.70)
	inter-spinosum	0.18–1.21	0.54 ± 0.22	0.53 (0.41–0.62)
	infra-spinosum	0.13–1.58	0.55 ± 0.20	0.52 (0.41–0.66)
Diameter at lingual terminal (mm)	supra-spinosum	0.30–1.97	0.87 ± 0.30	0.83 (0.65–1.03)
	inter-spinosum	0.38–1.69	0.76 ± 0.35	0.66 (0.55–0.91)
	infra-spinosum	0.18–2.06	0.80 ± 0.32	0.75 (0.59–0.95)

IQR: Inter quartile range; SD: Standard deviation.

**Table 3:** Comparison between males and females according to the number of canals and presence of supra, inter, and infra-spinosum canals (*n* = 320).

	Male (*n* = 160)		Female (*n* = 160)		*p* Value
	No.	%	No.	%	
Number of canals					<0.001 *
1	30	18.0	69	43.1	
2	96	60.0	78	48.8	
3	34	21.3	13	8.1	
Supra-spinosum					0.791
Present	152	95.0	153	95.6	
Absent	8	5.0	7	4.4	
Inter-spinosum					0.001 *
Present	34	21.3	13	8.1	
Absent	126	78.8	147	91.9	
Infra-spinosum					<0.001 *
Present	138	86.3	99	61.9	
Absent	22	13.8	61	38.1	

*: Statistically significant at *p* ≤ 0.05.

**Table 4:** Comparison between males and females according to study variables measurements (*n* = 320).

Variable	Category	Male (*n* = 160) (Mean ± SD)	Female (*n* = 160) (Mean ± SD)	*p* Value
Inferior border to buccal terminal (mm)	supra-spinosum	11.55 ± 2.49	10.51 ± 2.21	<0.001 *
	inter-spinosum	8.97 ± 2.0	6.53 ± 1.47	<0.001 *
	infra-spinosum	7.12 ± 2.06	5.52 ± 1.87	<0.001 *
Inferior border to lingual terminal (mm)	supra-spinosum	15.45 ± 2.25	14.44 ± 2.01	<0.001 *
	inter-spinosum	9.21 ± 2.47	7.45 ± 1.78	0.020 *
	infra-spinosum	5.30 ± 2.91	5.17 ± 2.43	0.85
Buccal cortex to buccal terminal (mm)	supra-spinosum	6.0 ± 1.59	5.69 ± 1.77	0.11
	inter-spinosum	6.29 ± 1.65	5.15 ± 1.61	0.033 *
	infra-spinosum	5.89 ± 1.65	5.69 ± 1.90	0.38
Length (mm)	supra-spinosum	6.18 ± 2.16	5.45 ± 1.93	0.006 *
	inter-spinosum	5.46 ± 1.73	5.65 ± 1.84	0.74
	infra-spinosum	5.40 ± 1.79	4.82 ± 1.91	0.015 *
Diameter at the buccal terminal (mm)	supra-spinosum	0.65 ± 0.19	0.55 ± 0.20	<0.001 *
	inter-spinosum	0.60 ± 0.21	0.41 ± 0.20	0.004 *
	infra-spinosum	0.58 ± 0.17	0.50 ± 0.22	<0.001 *
Diameter at the lingual terminal (mm)	supra-spinosum	0.84 ± 0.27	0.90 ± 0.33	0.233
	inter-spinosum	0.85 ± 0.37	0.55 ± 0.09	0.002 *
	infra-spinosum	0.84 ± 0.33	0.74 ± 0.30	0.008 *

*: Statistically significant at *p* ≤ 0.05; SD: Standard deviation.

**Table 5:** Correlation between length (mm) vs. diameter at the lingual terminal (mm) for supra-inter and infra spinosum canals.

	N	Length (mm) vs. Diameter at the Lingual Terminal (mm)	
		r_s_	*p*
Supra-spinosum	304	0.169 *	0.003 *
Inter-spinosum	48	0.139	0.345
Infra-spinosum	238	0.079	0.222

r_s_: Spearman coefficient; *: statistically significant at *p* ≤ 0.05.

## Data Availability

Data is available upon request from authors.

## References

[1] Woo B.M., Al-Bustani S., Ueeck B.A. (2006). Floor of Mouth Haemorrhage and Life-Threatening Airway Obstruction during Immediate Implant Placement in the Anterior Mandible. Int. J. Oral Maxillofac. Surg..

[2] Goller Bulut D., Köse E. (2018). Available Bone Morphology and Status of Neural Structures in the Mandibular Interforaminal Region: Three-Dimensional Analysis of Anatomical Structures. Surg. Radiol. Anat..

[3] Alqutaibi A.Y., Kaddah A.F., Farouk M. (2017). Randomized Study on the Effect of Single-Implant versus Two-Implant Retained Overdentures on Implant Loss and Muscle Activity: A 12-Month Follow-up Report. Int. J. Oral Maxillofac. Surg..

[4] Alqutaibi A.Y., Esposito M., Algabri R., Alfahad A., Kaddah A.F., Farouk M., Alsourori A. (2017). Single vs. Two Implant-Retained Overdentures for Edentulous Mandibles: A Systematic Review. Eur. J. Oral Implantol..

[5] Liang X., Jacobs R., Lambrichts I., Vandewalle G. (2007). Lingual Foramina on the Mandibular Midline Revisited: A Macroanatomical Study. Clin. Anat..

[6] Kawai T., Asaumi R., Sato I., Yoshida S., Yosue T., Kawai T., Asaumi R., Yosue T., Sato I., Yoshida S. (2007). Classification of the Lingual Foramina and Their Bony Canals in the Median Region of the Mandible: Cone Beam Computed Tomography Observations of Dry Japanese Mandibles. Oral Radiol..

[7] Rosano G., Taschieri S., Gaudy J.F., Testori T., del Fabbro M. (2009). Anatomic Assessment of the Anterior Mandible and Relative Hemorrhage Risk in Implant Dentistry: A Cadaveric Study. Clin. Oral Implants Res..

[8] Mordenfeld A. (1997). Hemorrhage in the Floor of the Mouth During Implant. Int. J. Oral Maxillofac. Implants.

[9] Longoni S., Sartori M., Braun M., Bravetti P., Lapi A., Baldoni M. (2007). Lingual Vascular Canals of the Mandible: The Risk of Bleeding Complications during Implant Procedures. Implants Dent..

[10] Jacobs R., Mraiwa N.D., Gijbels F., Quirynen M. (2002). Appearance, Location, Course, and Morphology of the Mandibular Incisive Canal: An Assessment on Spiral CT Scan. Dentomaxillofac. Radiol..

[11] Aoun G., Nasseh I., Sokhn S., Rifai M. (2017). Lingual Foramina and Canals of the Mandible: Anatomic Variations in a Lebanese Population. J. Clin. Imaging Sci..

[12] Poovannan S., Sarumathi T. (2022). Prevalence and Anatomic Variations of Lingual Foramina and Lingual Canal in Anterior Mandible Using Cone Beam Computed Tomography—A Cross-Sectional Study. J. Indian Acad. Oral Med. Radiol..

[13] Tepper G., Hofschneider U.B., Gahleitner A., Ulm C. (2001). Computed Tomographic Diagnosis and Localization of Bone Canals in the Mandibular Interforaminal Region for Prevention of Bleeding Complications during Implant Surgery. Int. J. Oral Maxillofac. Implants.

[14] Babiuc I., Tarlungeanu I., Pauna M. (2011). Cone Beam Computed Tomography Observations of the Lingual Foramina and Their Bony Canals in the Median Region of the Mandible. Rom. J. Morphol. Embryol..

[15] Sheikhi M., Mosavat F., Ahmadi A. (2012). Assessing the Anatomical Variations of Lingual Foramen and Its Bony Canals with CBCT Taken from 102 Patients in Isfahan. Dent. Res. J. Isfahan.

[16] Barbosa D.A.F., de Mendonça D.S., de Carvalho F.S.R., Kurita L.M., de Barros Silva P.G., Neves F.S., Costa F.W.G. (2022). Systematic Review and Meta-Analysis of Lingual Foramina Anatomy and Surgical-Related Aspects on Cone-Beam Computed Tomography: A Prospero-Registered Study. Oral Radiol..

[17] Choi D.Y., Woo Y.J., Won S.Y., Kim D.H., Kim H.J., Hu K.S. (2013). Topography of the Lingual Foramen Using Micro-Computed Tomography for Improving Safety during Implant Placement of Anterior Mandibular Region. J. Craniofac. Surg..

[18] Sanchez-Perez A., Boix-Garcia P., Lopez-Jornet P. (2018). Cone-Beam CT Assessment of the Position of the Medial Lingual Foramen for Dental Implant Placement in the Anterior Symphysis. Implants Dent..

[19] Lakha T., Kheur M., Mühlemann S., Kheur S., Le B. (2020). Ultrasound and CBCT Analysis of Blood Flow and Dimensions of the Lingual Vascular Canal: A Case Control Study. J. Oral Biol. Craniofac. Res..

[20] Mraiwa N., Jacobs R., Moerman P., Lambrichts I., van Steenberghe D., Quirynen M. (2003). Presence and Course of the Incisive Canal in the Human Mandibular Interforaminal Region: Two-Dimensional Imaging versus Anatomical Observations. Surg. Radiol. Anat..

[21] Yılmaz S., Calikoglu E.O., Kosan Z. (2017). For an Uncommon Neurosurgical Emergency in a Developing Country. Niger. J. Clin. Pract..

[22] Jasa G.R., Shimizu M., Okamura K., Tokumori K., Takeshita Y., Weerawanich W., Yoshiura K. (2017). Effects of Exposure Parameters and Slice Thickness on Detecting Clear and Unclear Mandibular Canals Using Cone Beam CT. Dentomaxillofac. Radiol..

[23] Oettlé A.C., Fourie J., Human-Baron R., Zyl A.W. (2013). The Midline Mandibular Lingual Canal: Importance in Implant Surgery. Clin. Implants Dent. Relat. Res..

[24] He P., Truong M.K., Adeeb N., Tubbs R.S., Iwanaga J. (2017). Clinical Anatomy and Surgical Significance of the Lingual Foramina and Their Canals. Clin. Anat..

[25] Martins J.N.R., Zhang Y., Zuben M., Vargas W., Seedat H.C., Santiago F. (2021). Worldwide Prevalence of a Lingual Canal in Mandibular Premolars: A Multicenter Cross-Sectional Study with Meta-Analysis. J. Endod..

[26] Katakami K., Mishima A., Kuribayashi A., Shimoda S., Hamada Y., Kobayashi K. (2009). Anatomical Characteristics of the Mandibular Lingual Foramina Observed on Limited Cone-Beam CT Images. Clin. Oral Res..

[27] Liang X., Jacobs R., Corpas L.S., Semal P., Lambrichts I. (2009). Chronologic and Geographic Variability of Neurovascular Structures in the Human Mandible. Forensic Sci. Int..

[28] Tagaya A., Matsuda Y., Nakajima K., Seki K., Okano T. (2009). Assessment of the Blood Supply to the Lingual Surface of the Mandible for Reduction of Bleeding during Implant Surgery. Clin. Oral Implants Res..

[29] von Arx T., Matter D., Buser D., Bornstein M.M. (2011). Evaluation of Location and Dimensions of Lingual Foramina Using Limited Cone-Beam Computed Tomography. J. Oral Maxillofac. Surg..

[30] He X., Jiang J., Cai W., Pan Y., Yang Y., Zhu K., Zheng Y. (2016). Assessment of the Appearance, Location and Morphology of Mandibular Lingual Foramina Using Cone Beam Computed Tomography. Int. Dent. J..

[31] Nimigean V., Sîrbu V.D., Nimigean V.R., Bădiţă D.G., Poll A., Moraru S.A., Păun D.L. (2018). Morphological Assessment of the Mandibular Canal Trajectory in Edentate Subjects. Rom. J. Morphol. Embryol..

[32] Taschieri S., Corbella S., Silnovic A., Francetti L., Messina C., Sconfienza L.M., Albano D. (2022). Frequency and Anatomic Variability of the Mandibular Lingual Foramina: A Cone-Beam CT Study. BMC Med. Imaging.

